# Supplementary material for: The causal relationship between the human gut microbiota and pyogenic arthritis: a Mendelian randomization study
Source: Front Cell Infect Microbiol. 2024 Nov 26;14:1452480. doi: 10.3389/fcimb.2024.1452480 (PMC11629706; doi:10.3389/fcimb.2024.1452480)
Supplement: Supplementary file 1 [file DataSheet1.zip › mendelian test/ebi-a-GCST90027631.csv_forest.pdf]

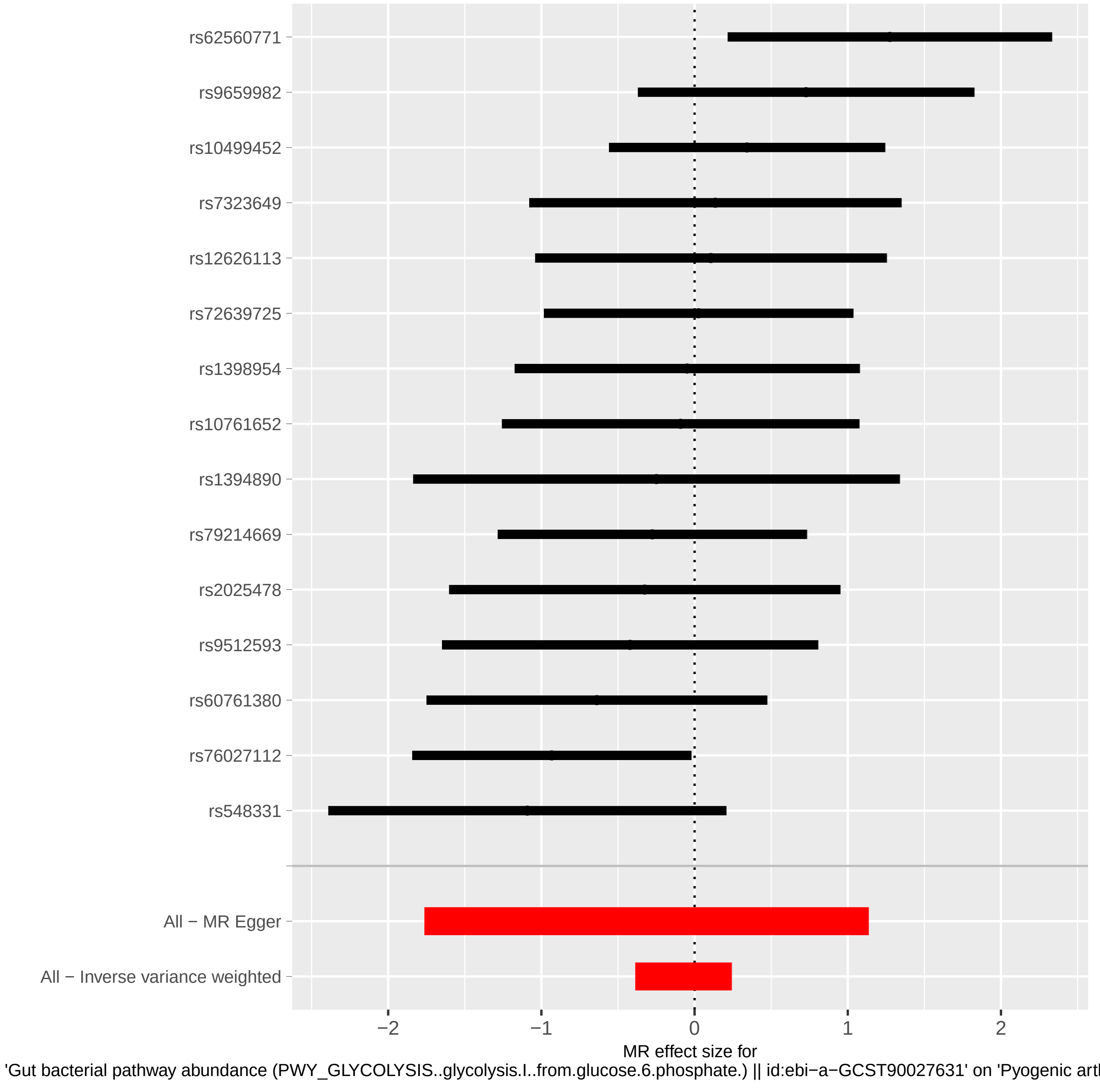

rs62560771
rs9659982
rs10499452
rs7323649
rs12626113
rs72639725
rs1398954
rs10761652
rs1394890
rs79214669
rs2025478
rs9512593
rs60761380
rs76027112
rs548331
All − MR Egger
All − Inverse variance weighted
−2
−1
0
1
2
MR effect size for
'Gut bacterial pathway abundance (PWY_GLYCOLYSIS..glycolysis.I..from.glucose.6.phosphate.) || id:ebi−a−GCST90027631' on 'Pyogenic art
